# Textile emitter for AI-enhanced human–machine interaction

**DOI:** 10.1093/nsr/nwag231

**Published:** 2026-04-17

**Authors:** Ya Sun, Xuanyu Cui, Gaoyang Kong, Jiaxin Li, Xiaomei Chen, Jianing Xu, Chengxin Xu, Peng Jin, Tao Hou, Hui Pan, Di Zhang, Han Zhou

**Affiliations:** State Key Laboratory of Metal Matrix Composites, School of Materials Science and Engineering, Shanghai Jiao Tong University, Shanghai 200240, China; Future Materials Innovation Center, Zhangjiang Institute for Advanced Study, Shanghai Jiao Tong University, Shanghai 201203, China; State Key Laboratory of Metal Matrix Composites, School of Materials Science and Engineering, Shanghai Jiao Tong University, Shanghai 200240, China; Future Materials Innovation Center, Zhangjiang Institute for Advanced Study, Shanghai Jiao Tong University, Shanghai 201203, China; State Key Laboratory of Metal Matrix Composites, School of Materials Science and Engineering, Shanghai Jiao Tong University, Shanghai 200240, China; Future Materials Innovation Center, Zhangjiang Institute for Advanced Study, Shanghai Jiao Tong University, Shanghai 201203, China; Department of Electrical and Computer Engineering, National University of Singapore, Singapore 117583, Singapore; Department of Electrical and Computer Engineering, National University of Singapore, Singapore 117583, Singapore; State Key Laboratory of Precision Measurement Technology and Instruments, Department of Precision Instruments, Tsinghua University, Beijing 100084, China; State Key Laboratory of Metal Matrix Composites, School of Materials Science and Engineering, Shanghai Jiao Tong University, Shanghai 200240, China; Future Materials Innovation Center, Zhangjiang Institute for Advanced Study, Shanghai Jiao Tong University, Shanghai 201203, China; State Key Laboratory of Robotics and System, Harbin Institute of Technology, Harbin 150001, China; Department of Electrical and Computer Engineering, National University of Singapore, Singapore 117583, Singapore; Department of Physics, State Key Laboratory of Surface Physics, and Key Laboratory of Micro and Nano Photonic Structures (MOE), Fudan University, Shanghai 200438, China; Department of Physics, Xiamen University, Xiamen 361005, China; State Key Laboratory of Metal Matrix Composites, School of Materials Science and Engineering, Shanghai Jiao Tong University, Shanghai 200240, China; State Key Laboratory of Metal Matrix Composites, School of Materials Science and Engineering, Shanghai Jiao Tong University, Shanghai 200240, China; State Key Laboratory of Metal Matrix Composites, School of Materials Science and Engineering, Shanghai Jiao Tong University, Shanghai 200240, China; Future Materials Innovation Center, Zhangjiang Institute for Advanced Study, Shanghai Jiao Tong University, Shanghai 201203, China

**Keywords:** textile emitters, strain-responsive infrared emission, AI-enhanced human–machine interaction, wireless interaction, smart photonic textiles

## Abstract

Wearable textile interfaces hold significant promise for advanced human–machine interaction, yet realizing wireless, battery-free on-body signal generation without embedded circuitry in textiles remains a formidable challenge. Here, we present a textile emitter that enables precise interaction by directly transducing user-induced mechanical deformation into time-coded infrared (IR) thermal signals. Constructed from strain-responsive IR-emitting fibers, the textile emitter simultaneously functions as a programmable signal generator without embedded electronic circuits or power sources within the textile. Seamlessly integrated into everyday garments, it ensures stable performance while maintaining comfort and wearability. Inspired by Morse code, user motions are encoded as temporally structured IR signals and wirelessly decoded by machine learning algorithms, achieving up to 99.6% recognition accuracy across diverse interaction commands. This AI-enhanced textile emitter establishes a scalable and robust foundation for future wearable systems in information communication, robotic control, and digital interaction.

## Teaser

This work reports a battery-free textile emitter that converts human motions into wireless infrared signals, enabling seamless and precise human–machine interaction through AI decoding.

## INTRODUCTION

The integration of artificial intelligence (AI) into human–machine interactive interfaces has led to significant advancements in applications such as health monitoring [1–4], information communication [5–7], robotic control [8–10], and immersive technologies including virtual and augmented reality [11–13]. In most existing interactive systems, signal generation and transmission are achieved through active devices that rely on continuous power supplies and require users to carry external hardware, such as smartwatches, smartphones, buttons, or keyboards. Representative implementations include electronic fiber-based sensors operating via resistance [14–17], capacitance [18–21], triboelectricity [22–25], and piezoelectricity [26–28]. The dependence on external circuitry [29,30] or power sources [31] can restrict deployment in scenarios demanding long-term wearability, high portability, or limited energy availability. In parallel, optical approaches, including visible-light luminescent textiles [32–34] and camera-based gesture recognition systems [35,36], have been explored for contactless interaction. While visible-light luminescent textiles rely on visually perceptible optical signals, camera-based systems depend on optical imaging and illumination conditions, which may degrade performance under low-light environments and inherently raise privacy concerns associated with identity-revealing imaging [37]. Together, these limitations highlight the challenge of realizing interactive interfaces that are simultaneously wearable, privacy-conscious, and energy-autonomous (Table S1).

Textiles, as ubiquitous and inherently flexible materials, offer natural conformity to the human body. Their softness, breathability, and wearability make them ideal candidates for next-generation interaction interfaces [38–42]. Despite these merits, the development of AI-compatible, wireless, and fully textile-based interfaces that operate without embedded power supplies remains limited, particularly for practical, real-world deployment. Achieving such interfaces requires signal-emitting mechanisms capable of translating human intent into machine-readable information while avoiding on-body electronic integration and preserving mechanical compliance and wearability. Infrared (IR) emission represents a promising alternative modality for wireless interaction, offering inherent advantages such as ambient light immunity, spatial confinement, and potential compatibility with energy-autonomous operation [43–45]. Nevertheless, the practical integration of IR-emissive functionalities into textile platforms suitable for interactive applications remains insufficiently developed.

**Figure 1.**
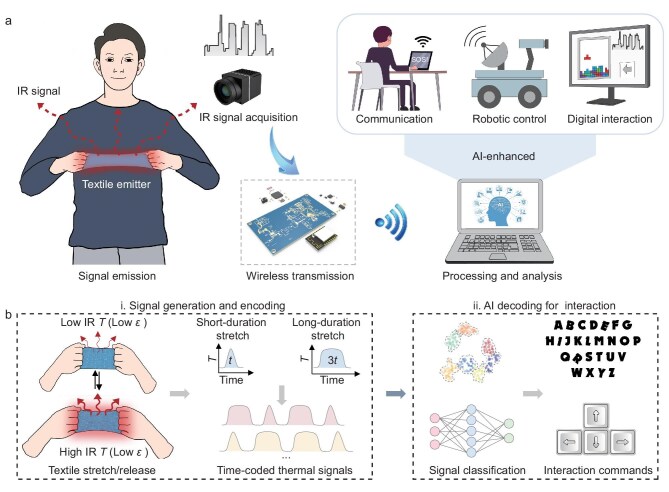
Conceptual illustration and working mechanism of the textile emitter for AI-enhanced information communication and human–machine interaction. (a) Schematic illustrating the interaction framework enabled by the textile emitter, including an on-body textile emitter for IR signal generation, off-body units for IR signal acquisition and transmission, and a smart terminal for AI-based signal decoding and analysis. The textile emitter responds to hand stretching and releasing motions by dynamically modulating its IR emissivity, thereby generating distinct IR thermal signals. These signals are captured and transmitted to the terminal, where machine learning algorithms decode the encoded patterns to enable interaction commands across diverse application scenarios, including information communication, robotic control, and digital interaction. (b) Working flow chart of the textile emitter for human–machine interaction. Mechanical stretching induces emissivity (*ε*) variations that generate corresponding IR thermal signals, where *T* represents temperature. By varying the stretching pause duration (*t*), time-coded IR thermal signal sequences are formed and classified by machine learning algorithms to output predefined interaction commands.

In this work, we developed a textile-based IR emitter for wireless and AI-enhanced human–machine interaction (Fig. 1a). The textile interface operates without embedded electronic circuits or power sources, enabling conformable integration into everyday garments. By leveraging strain-responsive emissivity, mechanical deformation induced by user motion is directly converted into modulated IR thermal signals. User actions are encoded into temporally structured thermal pulses following a time-domain encoding scheme inspired by Morse code principles, allowing deformation events to serve as machine-readable commands. These signals are decoded with machine learning algorithms, achieving recognition accuracies of up to 99.6% across multiple interaction commands. Our approach establishes a new class of textile-based energy-autonomous emissive interfaces, offering a scalable solution for intelligent, privacy-aware, and comfortable human–machine interaction.

## RESULTS AND DISCUSSION

### Conceptual design of the AI-enhanced textile emitter

We present a schematic illustration of the working principle underlying the textile emitter for information communication and command control (Fig. 1b). The textile emitter can be seamlessly integrated into everyday garments, responding to mechanical deformation such as hand stretching and converting these motions into modulated IR thermal signals (Fig. S1). In its undeformed state, the textile emitter exhibits intrinsically low emissivity, resulting in minimal IR emission and a correspondingly low apparent IR temperature when viewed through an IR temperature detector. Upon mechanical deformation, the emissivity increases sharply, producing a distinct rise in apparent IR temperature that can be detected in real time. Information is encoded using two types of IR thermal signals differentiated by stretch duration: short signals generated by rapid stretch–release, and long signals produced by sustained stretching. By combining these signals in different temporal sequences, time-coded thermal signal patterns corresponding to distinct interaction commands are formed.

For example, a rapid stretch–release motion yields a transient IR temperature pulse that can be interpreted as a ‘forward’ command, whereas sustained stretching for a defined duration before release produces a distinct thermal signal sequence corresponding to an alternative command, such as ‘left’. These encoded signals are captured by an IR temperature detector and subsequently transmitted wirelessly to smart processing terminals equipped with machine learning-based decoding algorithms. By extracting key features such as temporal frequency from the time-varying thermal signals, pre-trained machine learning models enable accurate mapping between physical deformations and predefined command outputs. Ultimately, users can interact with digital and robotic systems simply by manipulating the textile emitter embedded in their clothing, without relying on conventional input hardware such as buttons, screens, or rigid interfaces. Moreover, the localized nature of IR emission reduces unintended information exposure, providing an inherent advantage in privacy-sensitive environments.

### Working mechanism and signal emitting capability

**Figure 2.**
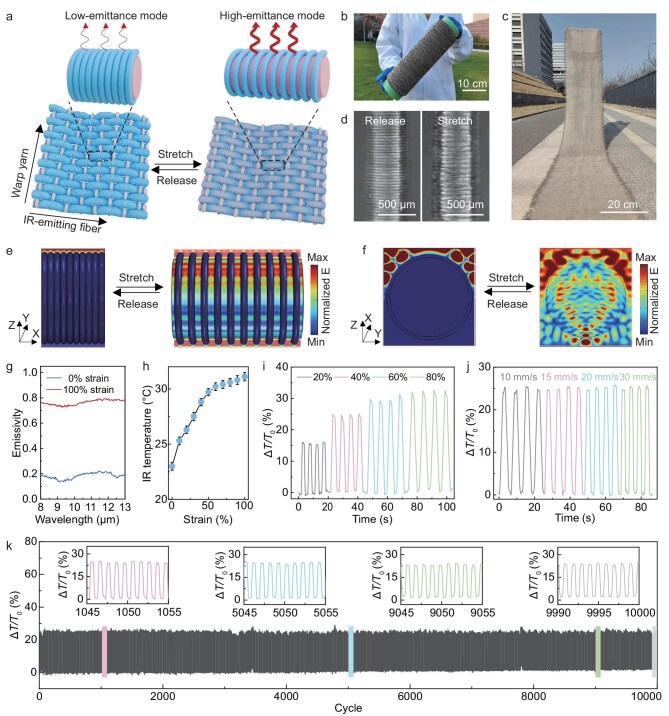
Working mechanism and signal emitting performance of the textile emitter. (a) Schematic illustration showing the structure and working mechanism of the textile emitter during the release and stretch states. The textile emitter is woven with strain-responsive IR-emitting fiber wefts and warp yarns. (b) Photograph of the strain-responsive IR-emitting fibers. (c) Photograph of the textile emitter fabricated over a large area, highlighting its potential for scalable production and practical wearable applications. (d) Photographs showing the structure change of the strain-responsive IR-emitting fiber in (left) released and (right) stretched states. (e, f) Electric field distributions in the X–Z plane (e) and Y–Z plane (f) of the strain-responsive IR-emitting fiber in released and stretched states. The incident light is at 10 μm. (g) Measured IR emissivity spectra of the textile emitter during release and stretch states. (h) Apparent IR temperature change with increasing strain. (i–k) Signal response (relative apparent IR temperature change) of the textile emitter under various conditions: (i) during stretch–release cycles to different strain levels, (j) during repeated stretch–release cycles to 40% strain at different actuation speeds, and (k) durability over 10 000 stretch–release cycles to 40% strain.

Figure 2a illustrates the design and mechanism of the textile emitter, which is constructed by weaving strain-responsive IR-emitting fibers and warp yarns. Each IR-emitting fiber consists of a co-wrapped heterostructure composite fiber, where low-emissivity silver (Ag) fibers helically wrap around high-emissivity polyurethane (PU) cores. In the release state, the Ag coils closely conform to the PU core, suppressing IR emission. Upon mechanical strain, the Ag coils separate, increasing the spacing between the Ag fiber and exposing the PU surface. This dynamic structural change enhances IR emissivity by modulating the interfacial geometry and optical pathways, enabling the textile to respond to mechanical strain and transduce it directly into IR emission for signal generation and encoding.

To enable scalable production, we fabricated kilometers of strain-responsive IR-emitting fiber using a co-wrap spinning method (Fig. 2b and Fig. S2), followed by weaving into meters of textile via an industrial rapier loom (Fig. 2c and Fig. S3). The resulting textile exhibits high mechanical flexibility and conforms to deformations such as twisting, stretching, and bending (Fig. S4), providing significant advantages over conventional rigid IR emitters for wearable applications. The woven architecture preserves intrinsic textile porosity, maintaining air permeability comparable to conventional apparel fabrics (Fig. S5). Morphological observations during deformation revealed increased interfiber spacing of the Ag coils under strain (Fig. 2d). In the initial unstretched state, adjacent coils are closely packed along the axial direction, whereas axial stretching increases the effective helical pitch and interfiber spacing, as illustrated in Fig. S6. Such strain-induced geometric reconfiguration gives rise to a strain-dependent modulation of IR emissivity (Fig. S7). To further elucidate this mechanism, numerical simulations of the electromagnetic field distribution were conducted, revealing a pronounced shift in electric field localization (Fig. 2e and f, Figs S8 and S9). In the unstretched state, the field was primarily concentrated on the surface, indicating high reflection by the outer Ag fiber. Upon stretching, the field redistributed toward the interior, indicating enhanced absorption within both the PU polymer core and the regions between fibers containing Ag coils. This structural modulation led to a significant enhancement in IR emissivity, increasing from 0.18 in the release state to 0.75 under 100% strain (Fig. 2g), accompanied by a continuous and tunable emissivity profile across the 8–13 μm atmospheric window (Fig. S10).

The textile emitter also demonstrated excellent mechanical robustness. Stress–strain testing revealed minimal hysteresis across different stretching speeds and repeated cycling (Fig. S11). Apparent IR temperature changes confirmed that IR emission intensity increased with strain (0%–100%) (Fig. 2h and Fig. S12), and remained detectable during different strains of stretching, facilitating intuitive interaction through simple user motions (Fig. 2i). The textile emitter maintained consistent signal output performance during repeated stretch–release cycles to 40% strain at different actuation speeds (Fig. 2j) and showed a characteristic response time of ∼667 ms corresponding to stretching from 0% to 40% strain under an actuation speed of 30 mm s^−1^ (Fig. S13). After 10 000 stretch–release cycles, the strain-dependent emissivity modulation remained stable, exhibiting an unchanged overall trend with only a slight reduction in modulation amplitude (Fig. S14). Consistently, the textile emitter maintained reliable signal output over 10 000 stretch–release cycles (Fig. 2k), with the relative apparent temperature change (*ΔT/T_0_*) remaining at ∼90% of its initial level. Thermal analysis, including differential scanning calorimetry and thermogravimetric analysis, indicated that the textile emitter exhibits excellent thermal stability (Fig. S15). The IR signal output remained robust under diverse environmental conditions, including different ambient temperatures (Fig. S16), 50 washing cycles, 200 h of UV aging, and 3 months of outdoor exposure (Fig. S17). These results validate the textile emitter’s robustness and practical viability for real-world wearable applications.

### Signal encoding and AI-enhanced information communication

**Figure 3.**
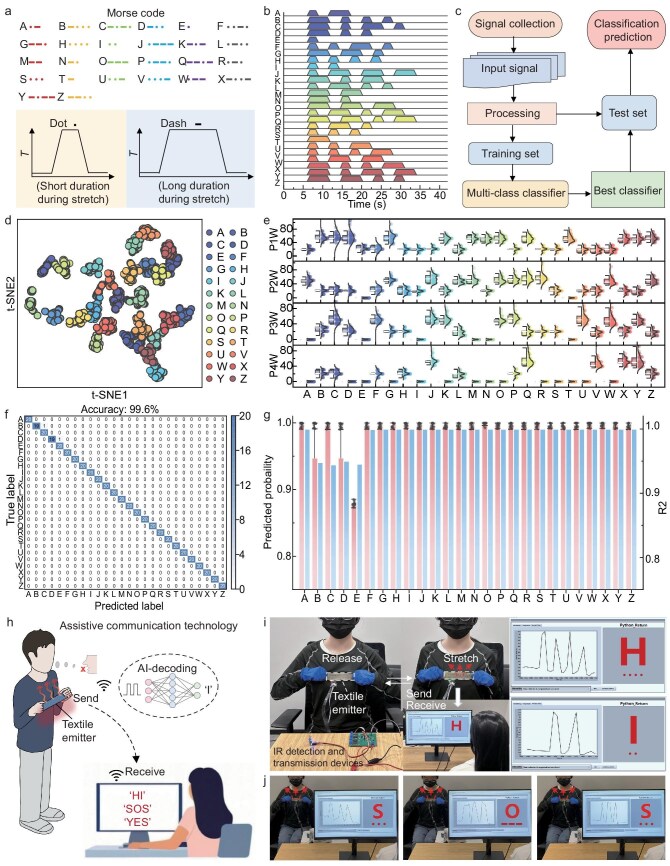
Signal encoding and AI-enhanced information communication using the textile emitter. (a) The 26 English letters and their Morse code representations (top), and thermal signal representations of the fundamental Morse code units (dot and dash), where short and long signal pulses are generated via rapid and sustained stretching, respectively (bottom). (b) Thermal signal sequences encoding the 26 letters based on Morse code using short and long signal pulses. (c) Algorithm flow chart showing the training and optimization process of the machine learning model for signal classification and prediction. (d) Two-dimensional t-SNE (t-distributed stochastic neighbor embedding) projection of the 26 English letters. (e) Extracted signal features, including peak count and widths (P1W–P4W), used for classification. P1W, P2W, P3W, and P4W refer to the widths of Peak 1, Peak 2, Peak 3, and Peak 4, respectively. (f) Confusion matrix showing classification results of the 26 English letters using the DT algorithm. (g) Prediction probabilities and corresponding coefficient of determination (*R*^2^) values for each letter using the DT algorithm. (h) Conceptual illustration of the textile emitter enabling message transmission through hand stretching. (i) Demonstration of the word ‘HI’ output using the textile emitter. (j) Emergency signaling example showing ‘SOS’ encoded and transmitted via the textile emitter.

The textile emitter reliably converts mechanical deformations into time-coded IR thermal signals, enabling temporally structured signal generation under repeated stretching actions. To demonstrate structured information transmission, an encoding framework inspired by Morse code was implemented (Fig. 3a, top). Two user-defined stretching patterns, characterized by short and long stretching durations prior to release, produce distinguishable thermal signal segments that represent ‘dot’ and ‘dash’ signals, respectively (Fig. 3a, bottom). The dot–dash distinction is based on the relative temporal structure of pulses, enabling stable discrimination under natural user-induced temporal variability (Fig. S18). By repeating and permuting these basic units, users can generate diverse thermal signal sequences corresponding to the 26 English letters, enabling encoded message transmission through intuitive stretching actions (Fig. 3b). The interaction process relies on passively emitted IR thermal signals generated by the textile emitter, thereby establishing a non-visual, privacy-conscious interaction modality.

The workflow of the machine learning-based decoding process, illustrated in Fig. 3c, enables reliable classification of temporally encoded IR thermal signals under realistic operating variability encountered in practical use, including inter-user deformation differences and environmental fluctuations. The dataset comprising ∼2600 thermal signal sequences (100 per letter) was collected and preprocessed through moving-average-based baseline computation, normalization, and length standardization (see Methods). Although the thermal-pulse amplitude (*ΔT*) may vary with certain actuation conditions (Fig. S19), the temporal structure of the pulse sequence remains consistent, enabling stable interpretation based on temporal features.

The dataset was then divided into training and testing subsets. A multiclass classification model was trained using 2080 samples, while the remaining 520 samples were reserved for validation. Five machine learning algorithms—random forest (RF), k-nearest neighbors (KNN), decision tree (DT), eXtreme Gradient Boosting (XGBoost), and support vector machine (SVM)—were evaluated to assess recognition performance and computational efficiency. The thermal signal profiles corresponding to each letter before and after processing exhibited strong consistency (Fig. S20). Dimensionality reduction using t-SNE revealed clear separability among the encoded signal classes (Fig. 3d). Key signal features, including peak count and peak width, served as effective discriminative markers for classification (Fig. 3e, Figs S21 and S22).

The confusion matrices and area under the curve obtained using five classification algorithms demonstrated high recognition performance across all 26 classes (Fig. 3f, Figs S23 and S24). Recognition accuracies of 93.3% for KNN, 98.8% for SVM, 99.6% for both DT and RF, and 96.5% for XGBoost were achieved (Fig. S25). Precision, recall, and F1-score analyses further confirmed balanced classification performance for all evaluated models (Fig. S26). Among these algorithms, the DT model was selected for subsequent deployment due to its favorable balance between classification accuracy and computational efficiency. This can be attributed to the low dimensionality and strong discriminative nature of the extracted features, which encode rule-like temporal patterns that align well with DT’s threshold-based splitting mechanism. The prediction outputs from the DT algorithm exhibited tightly clustered probability distributions and high *R*^2^ values across all 26 classes, indicating reliable and consistent decoding performance (Fig. 3g). The recognition performance remained stable within a practical working range of IR detector distances and viewing angles (Fig. S27), as well as under diverse ambient conditions (Fig. S28).

To further demonstrate practical feasibility, the trained classification model was integrated into a wearable communication prototype. The textile emitter was embedded in the chest region of a garment (Fig. 3h), allowing the user to generate encoded signals through stretching motions. The emitted IR thermal signals were wirelessly captured by an IR temperature detector and transmitted via a microcontroller to a smart terminal for decoding (Fig. S29). The terminal decoded the incoming signal using the pre-trained model and displayed the corresponding text on a computer interface. As a proof of concept, the message ‘HI’ was successfully transmitted using this protocol (Fig. 3i and Video S1). Additional messages such as ‘SOS’ and ‘YES’ were similarly encoded and decoded (Fig. 3j, Fig. S30, and Videos S2 and S3), demonstrating the potential of this system for silent communication, particularly beneficial for individuals with speech impairments or in privacy-sensitive environments.

### Demonstrations of robotic vehicle control and digital interaction

**Figure 4.**
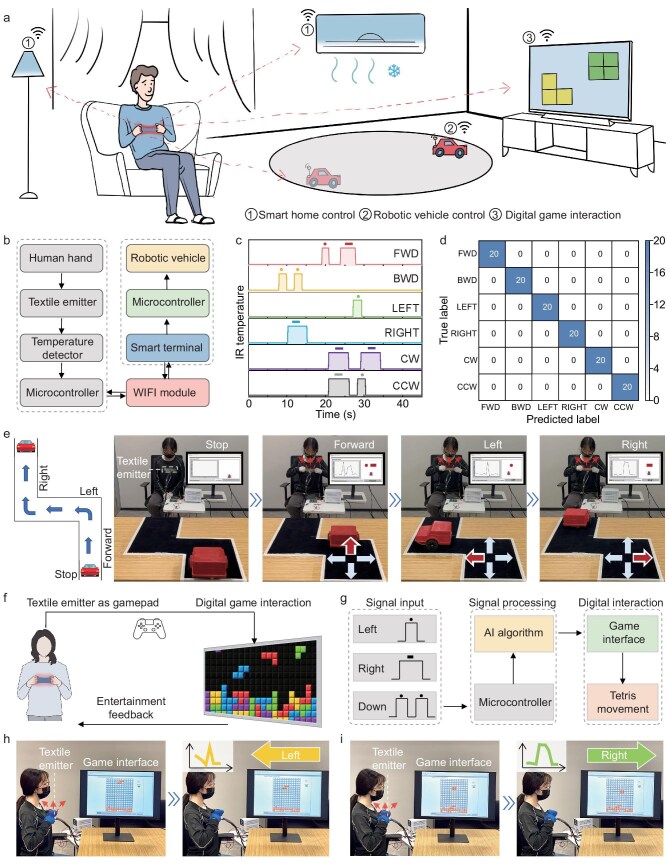
Demonstration of the textile emitter for wireless robotic vehicle control and digital game interaction. (a) Schematic illustration of the textile emitter used as a wearable interface for generating temporally encoded IR thermal signals, which are decoded and translated into control commands. (b) Block diagram for wireless robotic vehicle control. (c) Thermal signal outputs of the textile emitter corresponding to six control commands: forward (FWD), backward (BWD), left, right, clockwise (CW), and counterclockwise (CCW). (d) Confusion matrix showing classification accuracy for the six control commands using the DT algorithm. (e) Demonstration of robotic vehicle control via the textile emitter. (f) Schematic of interaction with a digital game environment enabled by the textile emitter. (g) Workflow illustrating the wireless game interaction process. (h, i) Demonstrations of wireless game control realized via the textile emitter.

To demonstrate the practical utility of the textile emitter in smart living environments, we developed a wearable interactive prototype in which the textile emitter is integrated into garments and operated through simple stretching motions. As illustrated in Fig. 4a, users wearing clothing embedded with the textile emitter can generate IR thermal signals through mechanical deformation of the textile, without embedding electronic circuits or power sources within the textile itself. This concept was first validated through the manipulation of a robotic vehicle. Stretch-induced IR thermal signals were wirelessly captured, transmitted via WiFi, decoded using machine learning-based algorithms, and translated into corresponding actuation commands for vehicle control (Fig. 4b).

Leveraging the Morse code-inspired communication protocol, we implemented a dot-and-dash encoding scheme to define six distinct control commands: forward (FWD), backward (BWD), left, right, clockwise (CW), and counter-clockwise (CCW) (Fig. S31). Each stretch–release action produced a characteristic thermal signal, captured by IR temperature detector and processed to reveal distinct amplitude and timing features (Fig. 4c and Fig. S32). These thermal signals were classified with 100% accuracy using a DT algorithm (Fig. 4d and Fig. S33), ensuring the reliable interpretation of user intent and the reliable execution of commands. A robotic vehicle was used as a demonstration platform to verify the precise control capability of the textile emitter (Fig. 4e and Video S4). Upon completing a stretch–release action, the corresponding IR thermal signal was transmitted, decoded, and converted into movement commands such as ‘forward’, ‘left’, or ‘right’. This system enabled reliable and precise control using natural, body-based input. Moreover, the encoding scheme supports flexible expansion, allowing users to define custom commands based on specific application needs, highlighting the flexibility of the encoding scheme in human–machine interaction and assistive robotics. To demonstrate wireless interaction with a digital game environment, the wearable textile emitter was used as a flexible game-control interface for Tetris (Fig. 4f). Mechanical stretching and release of the textile generated distinct IR thermal signal patterns corresponding to three control commands (‘left’, ‘right’, and ‘down’) (Fig. S34). These signals were decoded and translated into game actions in real time (Fig. 4g). Using the DT algorithm, all three control commands were consistently recognized with 100% accuracy (Figs S35 and S36), enabling precise execution of the corresponding game actions. As shown in Fig. 4h and i, users were able to manipulate game elements using different stretch–release actions, illustrating the feasibility of using a textile-based interaction interface as an input modality for digital interaction (Video S5). These demonstrations highlight the suitability of the textile emitter as a passive and non-visual interaction interface for wearable applications. As a radiative interaction modality, effective operation relies on a clear thermal radiation path between the textile emitter and the detector, making it suited for body-coupled interaction scenarios.

## CONCLUSION

In summary, we developed a textile emitter that enables mechanically programmable thermal signal generation for wireless human–machine interaction. By leveraging strain-responsive IR-emitting fibers, the textile emitter converts mechanical deformation induced by user motion into time-coded IR thermal signals, allowing interaction without embedding electronic circuits or power sources within the textile. This textile-level energy-autonomous architecture supports conformable integration into everyday garments and intuitive, on-body operation. Coupled with machine learning-based decoding, the system enables reliable recognition of user-defined commands, demonstrating its applicability in information communication, robotic control, and digital interactive scenarios. The work offers a promising pathway toward wearable interactive systems for next-generation communication, autonomous interfaces, and embodied intelligent technologies.

Looking forward, the proposed emissive textile interaction paradigm may be integrated with other wearable sensing modalities, such as physiological or motion-related sensors, to enable multimodal systems for health-related monitoring and human–machine interaction. In addition, the non-visual and non-imaging nature of IR-based communication offers advantages for privacy-preserving interaction scenarios, motivating further exploration of system-level integration in future wearable interaction systems.

## METHODS

Detailed methods can be found in the online supplementary information.

## ETHICAL STATEMENT

Consent was obtained to publish identifiable images of study participants. All data were collected with the informed consent of each participant.

## Supplementary Material

nwag231_Supplemental_Files
