# Supplementary material for: Textile emitter for AI-enhanced human–machine interaction
Source: Natl Sci Rev. 2026 Apr 17;13(12):nwag231. doi: 10.1093/nsr/nwag231 (PMC13296564; doi:10.1093/nsr/nwag231)
Supplement: nwag231_Supplemental_Files [file nwag231_supplemental_files.zip › Supplementary data.pdf]

Supplementary Materials for

Textile emitter for AI-enhanced human–machine interaction

Ya Sun[1,2,†], Xuanyu Cui[1,2,†], Gaoyang Kong[1,2], Jiaxin Li[3], Xiaomei Chen[3,4], Jianing Xu[1,2], Chengxin Xu[5], Peng Jin[3,6], Tao Hou[7], Hui Pan[1], Di Zhang[1] and Han Zhou[1,2,*]

[1]State Key Laboratory of Metal Matrix Composites, School of Materials Science and Engineering, Shanghai Jiao Tong University, Shanghai 200240, China;

[2]Future Materials Innovation Center, Zhangjiang Institute for Advanced Study, Shanghai Jiao Tong University, Shanghai 201203, China;

[3]Department of Electrical and Computer Engineering, National University of Singapore, Singapore 117583, Singapore;

[4]State Key Laboratory of Precision Measurement Technology and Instruments, Department of Precision Instruments, Tsinghua University; Beijing, 100084, China;

[5]State Key Laboratory of Robotics and System, Harbin Institute of Technology, Harbin 150001, China;

[6]Department of Physics, State Key Laboratory of Surface Physics, and Key Laboratory of Micro and Nano Photonic Structures (MOE), Fudan University, Shanghai 200438, China;

[7]Department of Physics, Xiamen University, Xiamen, 361005, China

*Corresponding author. Email: hanzhou_81@sjtu.edu.cn

†Equally contributed to this work.

Supplementary Text

METHODS

Fabrication of strain-responsive IR-emitting fiber

The strain-responsive IR-emitting fiber was fabricated using a self-refitted, custom-made co-wrap spinning apparatus (Hengrun Textile Machinery Co., Ltd.). The PU fibers (500 μm, Dongguan Yunze Garment Accessories Co., Ltd.) were selected as the inner core fibers and the Ag fibers (10D, Jinan Yumo Science and Trade Co., Ltd.) were selected as the outer shell fibers. The wrapping speed of Ag fibers and drawing speed of PU fibers were 1400 rpm and 52 mm min^{-1}, respectively. After co-wrap spinning, the Ag fibers were continuously and uniformly wrapped around the PU fibers, forming heterostructure composite fibers.

Fabrication of the textile emitter

The textile emitter was woven by the above IR-emitting fibers as weft and Ag fibers as warp, using a semi-automatic industrial rapier loom (Y208W, Jiangyin Tongyuan Textile Machinery Co., Ltd.). The lifting plan designed for the plain fabric structure was input into the control computer of the loom for the weaving process.

Fabrication of the whole interactive system for application demonstration

The whole system for smart interaction comprised the textile emitter, an IR temperature detector, a microcontroller, a WiFi module, and smart terminals equipped with machine learning algorithms. All electronic components were located off-body or external to the textile emitter. For the interaction application, a piece of the textile emitter was affixed to a commercial quick-dry garment using 3M adhesive or sewing. An IR temperature detector (MLX906) continuously monitored the apparent IR temperature of the textile. A microcontroller (STM32) processed the thermal data and transmitted it wirelessly to a smart terminal via a WiFi module (ESP8266). The time-coded thermal signals were

displayed in real time and simultaneously subjected to preprocessing in a Python-based interface. These standardized signals were then input into a pre-trained machine learning model for classification. The resulting predictions were visualized through a LabVIEW-based graphical user interface, which concurrently triggered interactive commands for human–machine interface control.

Numerical simulations

The electromagnetic response of the IR-emitting fiber under stretching and releasing state was simulated using COMSOL. The optical properties (refractive index and extinction index) of Ag fibers and PU fibers were obtained from open-access refractive index databases. The simulations were conducted within a wavelength range of 8-13 μm. Perfectly matched layers (PMLs) were applied along the direction of light propagation, while periodic boundary conditions were used in the transverse directions.

Characterizations

The morphology and structure of the IR-emitting fibers were characterized by scanning electron microscopy (SEM, JSM-7800). The optical images of the IR-emitting fibers and the textile emitter were taken using a Nikon D610. The IR thermal images were taken using an IR camera (FLIR T630). The IR reflectance and transmittance spectra were measured by a Fourier transform infrared spectrometer (Nicolet iS50, Thermo Scientific) equipped with an IR integrating sphere (IntegratIR, Peak Technologies). The IR emissivity was calculated by 1 − reflectance − transmittance. The loading/unloading stress–strain curves were measured by a dynamic thermomechanical analyzer (DMA 850) at room temperature. DSC was carried out using DSC 250 (TA Instruments) thermal analyzers in a nitrogen atmosphere with temperatures from −50 to 200 °C (10°C min^{-1}). TGA was conducted using a TGA 550 tester (TA Instruments) over a temperature range from room temperature to 800 °C.

Air permeability test

The air permeability of the textile samples was measured using an air permeability instrument (YG461E, Wenzhou Fangyuan Instrument Co., Ltd., China), in accordance with GB/T 24218.15-2018.

Washing stability test

The textile emitter was washed in a household washing machine (NJR-XP10) with 50 washing and drying times, following the ISO 6330:2012 standard procedure. After 50 washing cycles, we measured signal response (relative apparent IR temperature changes) of the textile emitter for comparison.

UV aging stability test

The accelerated UV aging test of the textile emitter was conducted in a custom-built chamber with an UVA lamp (100 W m^{-2}, UVA-340) at 60 °C for 200 hours, which is equivalent to one year of sun exposure in Shanghai (annual UV dose of 64.84 kWh m^{-2}).

Outdoor weather-resistant stability test

To evaluate the outdoor durability, the textile emitter was placed in an open-roof outdoor scene (Shanghai, China) with long-term direct outdoor exposure to various weather conditions outdoors (e.g., sunny, rainy, and cloudy) for approximately 3 months (from 15 February 2024 to 15 May 2024).

Data acquisition, processing and feature engineering

The IR thermal signal database was acquired using a motorized tensile stage operated under displacement control. This controlled actuation ensured repeatable and well-

defined deformation conditions during data acquisition, which is essential for reproducible characterization of strain-dependent thermal signal patterns. The applied strain was calculated based on the programmed displacement relative to the initial gauge length of the textile emitter. The achievable strain measurement accuracy, primarily limited by the displacement resolution of the actuator and the definition of the initial gauge length, is estimated to be on the order of ±1%. Accordingly, the attainable strain precision of the present setup, defined by the repeatability of displacement-controlled actuation, is at the percent level. Unless otherwise specified, motorized actuation was used for quantitative data acquisition and analysis, while manual deformation was employed only for interaction demonstrations.

For each encoded signal, we conducted 100 sampling repetitions, accumulating a large amount of time-series temperature data. These raw data were normalized using max-min scaling to constrain values within the [0, 1] interval while preserving the original data distribution. Each sample was uniformly processed to a fixed length of 750 sampling points via padding or truncation. Subsequently, adaptive binarization was applied to convert the data into spike signals. A moving average method with a window size of 75 was employed to dynamically compute the baseline, mitigating short-term fluctuations. A fixed offset of 0.5 was incorporated to establish adaptive thresholds for each sample, defined as the sum of the dynamic baseline and the offset. A spike event (assigned a value of 1) was identified when the signal continuously exceeded the adaptive threshold for five consecutive sampling points; otherwise, the value was set to 0. From the generated binary (0–1) sequences, all contiguous ‘1’ intervals were detected, and the rising (0→1) and falling (1→0) edges were localized. Based on these edge positions, the start/end points and widths of each spike were calculated. Two features—the number of spikes and their corresponding widths—were extracted for each sample.

Machine learning modeling

Five machine learning algorithms-KNN, RF, DT, SVM, and XGBoost-were implemented. Hyperparameter optimization for each model was conducted via

randomized search combined with five-fold cross-validation to ensure robust generalization. The average performance metrics across folds were reported to evaluate model efficacy. This systematic approach balanced computational efficiency and predictive accuracy while mitigating overfitting risks.

Methods for wireless information communication

The IR temperature detector captured thermal signal variations of different durations generated by the textile emitter. Within the LabVIEW interface, clicking the ‘Start’ button initiated a TCP/IP connection to the thermal matrix detector and began streaming temperature data. Data frames were read from the buffer until empty bytes were detected, marking completion. Upon triggering the ‘Complete & Calculate’ function, the program invoked a Python module and transferred the average temperature data for further processing. The data underwent normalization and adaptive binarization, followed by feature extraction. A pre-trained machine learning model then classified the processed signals, identifying one of the 26 English letters. The result was returned to LabVIEW and displayed in the user interface. This integrated system enabled effective communication via fabric manipulation by seamlessly linking thermal signal acquisition, intelligent classification, and user feedback.

Methods for wireless robotic vehicle control

Following the methods for wireless information communication, the acquired data underwent preprocessing before being input into a pre-trained machine learning model. The model computed one of six control commands- ‘Forward’, ‘Backward’, ‘Left’, ‘Right’, ‘Clockwise’, or ‘Counter-clockwise’, and the classified command was visualized within the program interface. Upon receiving the output string from Python-based interface, the system routed the command to a dedicated vehicle control thread. This thread transmitted the instruction via Bluetooth serial interface to the physical vehicle, actuating the corresponding movement. Simultaneously, the LabVIEW interface constructed a simulated vehicle coordinate mapping model, which

synchronized with real-world motions through position tracking algorithms. This implementation demonstrated an integrated workflow combining thermal signal interpretation, intelligent decision-making, reliable physical actuation, and virtual-physical synchronization, demonstrating a cohesive cyber-physical system for human-robot interaction via textile-based control interfaces.

Methods for digital game interaction

Following the methods for wireless information communication described above, the acquired data underwent preprocessing before being input into a pre-trained machine learning model. The model computed one of three control commands: ‘down’, ‘left’, or ‘right’. Upon receiving the classified output string from a Python-based interface, the program integrated it into a Tetris gaming module. This string directly governed in-game actions, executing either a leftward shift, rightward shift, or accelerated downward movement of Tetris elements within the game interface. This implementation demonstrated an integrated workflow combining thermal signal processing, machine learning classification, and game control through textile-based human-machine interaction.

Figures

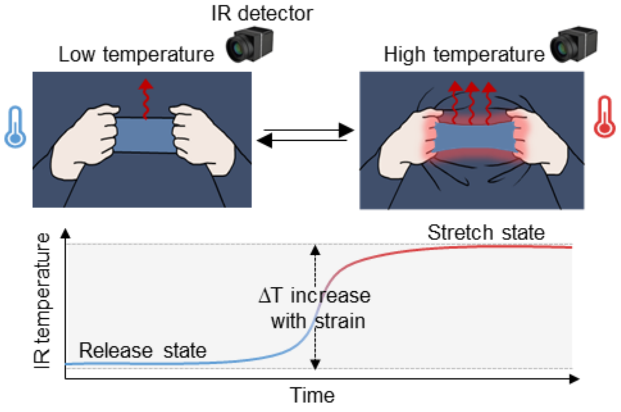

Figure S1. Schematic illustration of signal emission of the textile emitter. In the released state, the textile emitter exhibits low emissivity, resulting in a weak IR signal with a relatively low apparent IR temperature. Upon stretching, the emissivity increases due to structural changes, leading to a significant rise in the apparent IR temperature. This strain-dependent response enables the textile emitter to act as a dynamic signal generator that emits thermal signals corresponding to mechanical deformation.

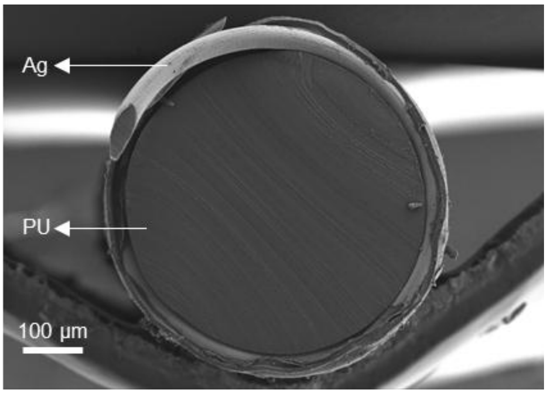

Figure S2. SEM images showing the cross-section of a strain-responsive IR-emitting fiber. The Ag fiber is tightly wrapped around the PU fiber to form a regular core-shell structure. The fiber radii of Ag fiber and PU fiber is around 18 and 250 μm, respectively.

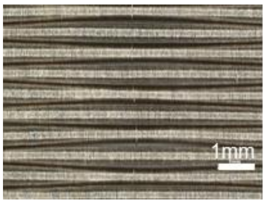

Figure S3. Optical image of the textile emitter showing its woven construction.

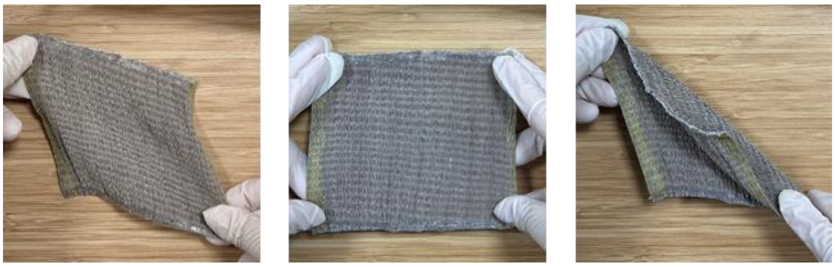

Figure S4. Photographs showing the textile emitter under twisting, stretching, and bending deformations.

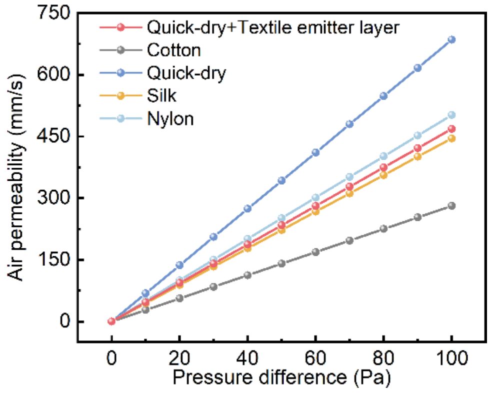

Figure S5. Air permeability of the textile-emitter-integrated garment compared with representative commercial apparel fabrics.

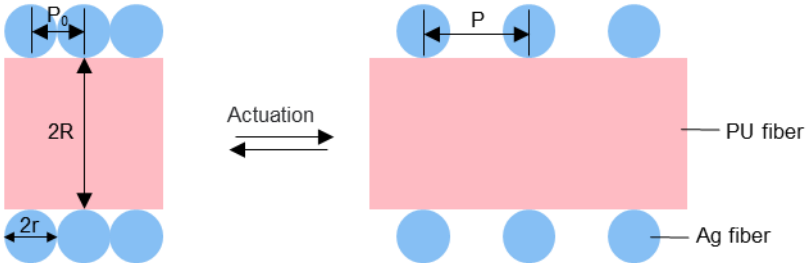

Figure S6. Schematic illustration of strain-induced geometric evolution of the IR-emitting fiber. In the initial undeformed state, adjacent Ag coils are tightly packed along the axial direction, such that the initial pitch (P_0) is approximately equal to the coil diameter ($P_0 \approx 2r$). Upon axial stretching, the helical structure undergoes an increase in pitch ($P_0 \rightarrow P$), leading to a reconfiguration of the coil spacing and effective metallic distribution. This schematic serves to define the key geometrical parameter (pitch) and to illustrate its monotonic evolution with applied strain.

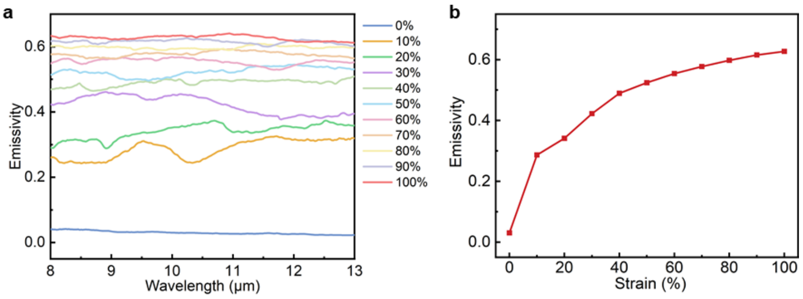

Figure S7. Simulated IR emissivity response of the IR-emitting fiber under different strains. (a) Simulated emissivity spectra in the IR range (8–13 μm) for different applied strains, modeled by varying the helical pitch according to strain-dependent geometrical configurations. (b) Corresponding emissivity as a function of applied strain, showing a gradual and monotonic increase in emissivity with increasing strain.

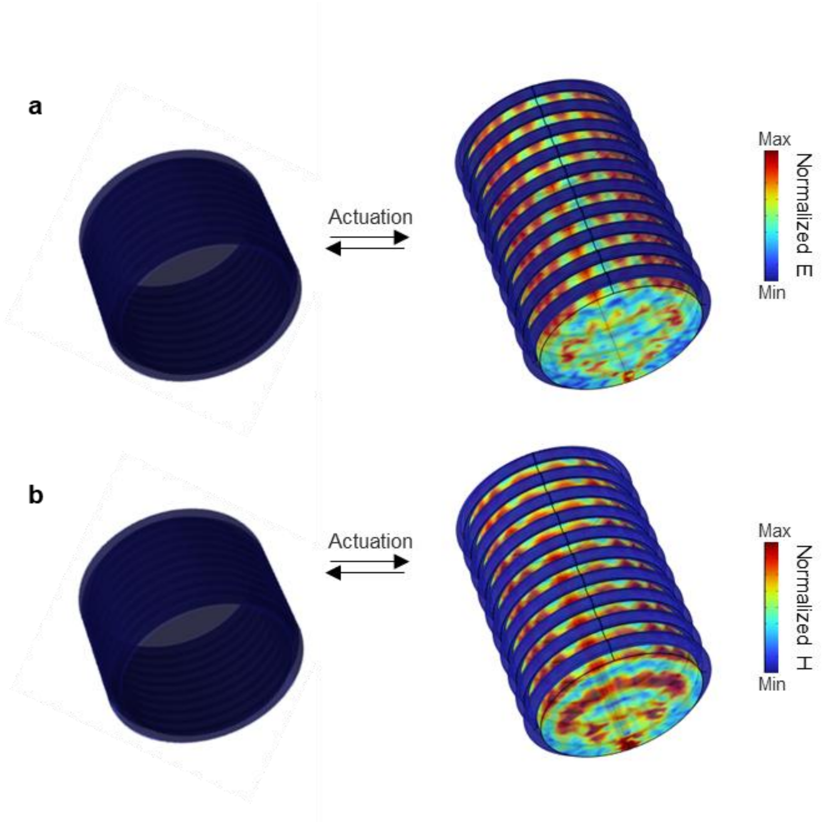

Figure S8. Simulated three-dimensional electromagnetic field distributions of the strain-responsive IR-emitting fiber before and after actuation. (a, b) Electric field (a) and magnetic field (b) distributions at a wavelength of 10 μm for the strain-responsive IR-emitting fiber before and after actuation.

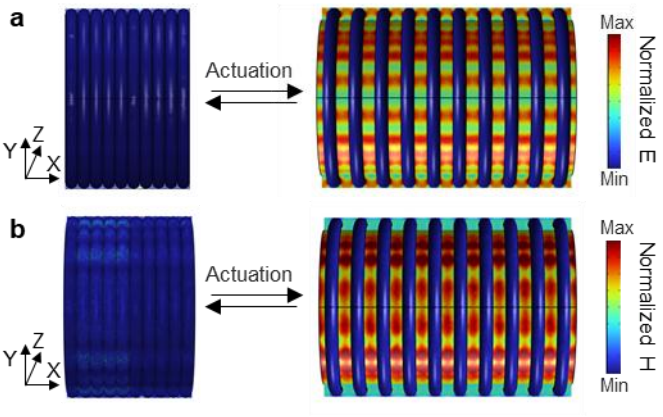

Figure S9. Electromagnetic field distributions of the strain-responsive IR-emitting fiber before and after actuation. (a, b) Electric field (a) and magnetic field (b) distributions in the X–Y plane at a wavelength of 10 μm for the strain-responsive IR-emitting fiber before and after actuation.

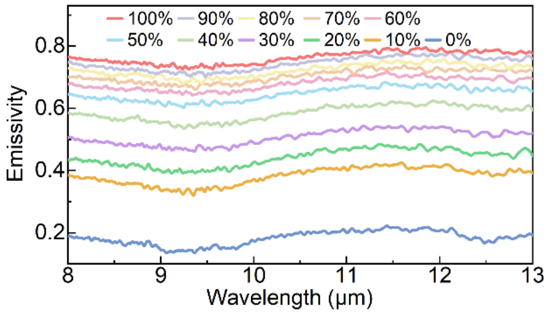

Figure S10. Experimental IR emissivity spectra of the textile emitter under different strains.

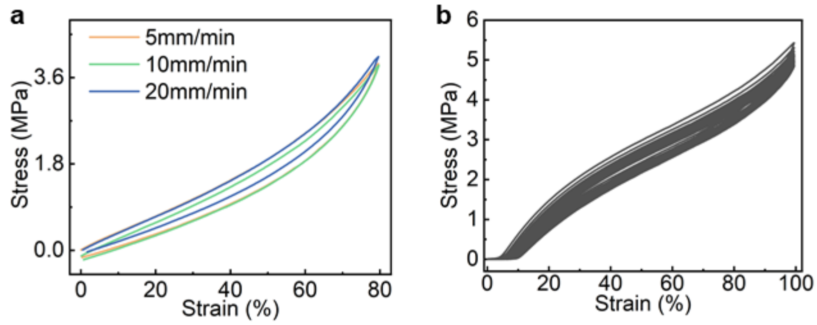

Figure S11. Mechanical response of the textile emitter under tensile deformation. (a) Loading and unloading curves at 80% strain under different speeds. (b) Cyclic loading and unloading curves at 100% strain over 20 cycles, showing stable mechanical behavior without noticeable degradation.

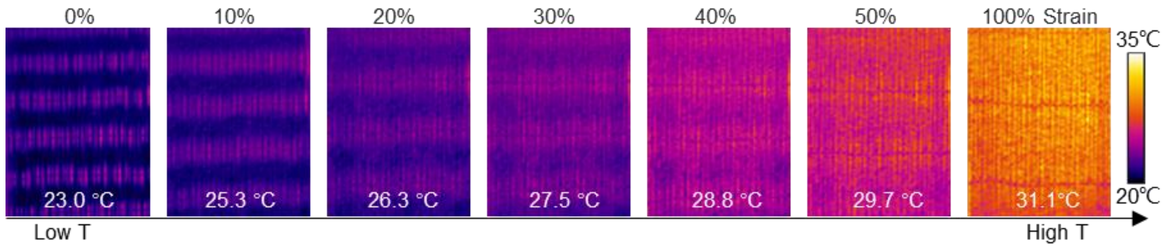

Figure S12. Thermal camera images of the textile emitter under different strains. As strain increases from 0% to 100%, the textile emitter exhibits a progressively enhanced thermal signal, accompanied by a clear increase in apparent IR temperature.

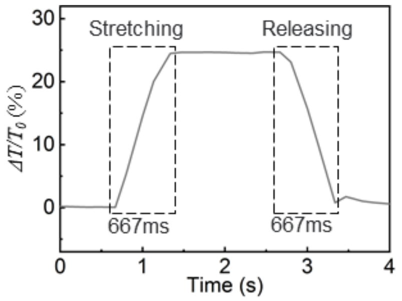

Figure S13. **Temporal evolution of the IR thermal signal during a representative stretch–release event to 40% strain at an actuation speed of 30 mm s^{-1}, illustrating stable and distinguishable signal generation under controlled mechanical deformation.**

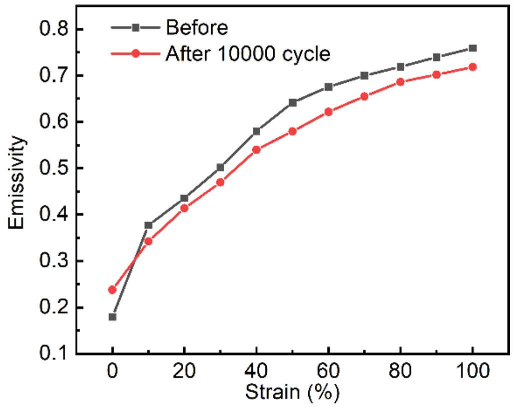

Figure S14. Emissivity–strain curves of the textile emitter measured before and after 10,000 stretch–release cycles.

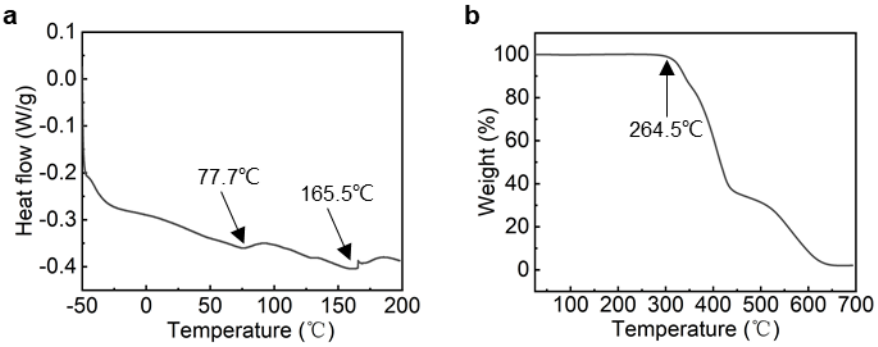

Figure S15. **Thermal properties and stability analysis of the textile emitter.** (a, b) DSC (a) and TGA (b) curves of the textile emitter, showing a glass transition temperature of 77.7 °C, a melting point of 165.5 °C, and a decomposition temperature of approximately 264.5 °C.

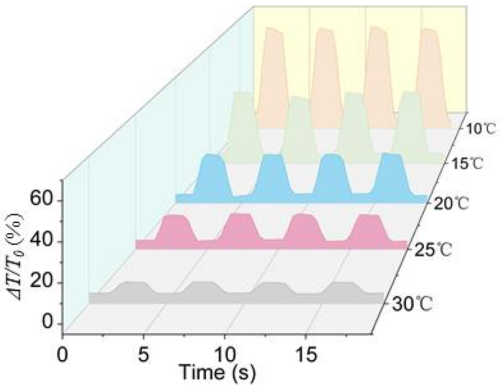

Figure S16. **Signal response of the textile emitter under different environmental temperatures.**

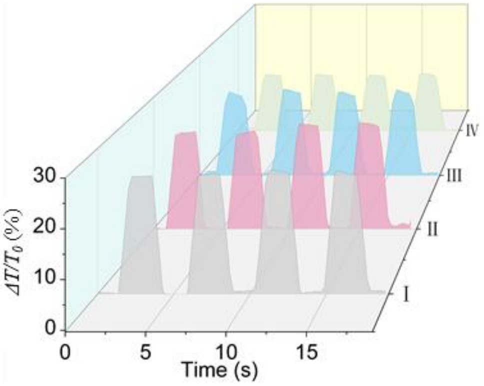

Figure S17. **Durability evaluation of the textile emitter under various environmental conditions.** Signal response of the textile emitter before (I) and after undergoing 50 washing cycles (II), UV aging at 60 °C for over 200 hours (III), and outdoor exposure for 3 months (IV).

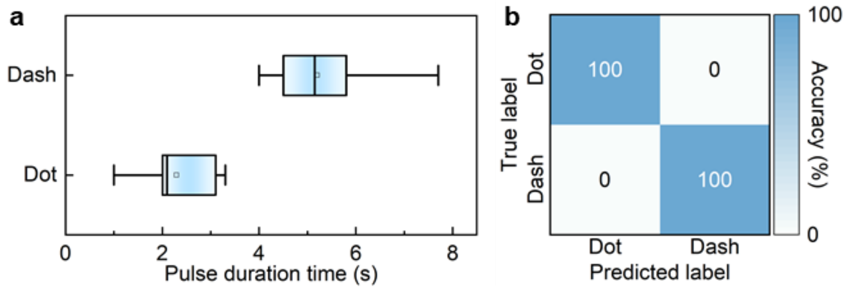

Figure S18. Temporal robustness of short- and long-pulse encoding across users. (a) Distributions of pulse durations for short (‘dot’) and long (‘dash’) pulses collected from six users with different operating habits, showing distinguishable duration ranges despite natural inter-user variability. (b) Confusion matrix for short- and long-pulse classification across six users.

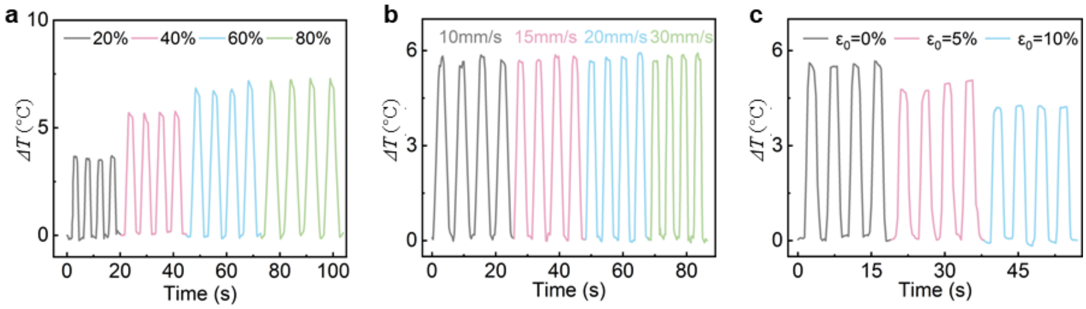

Figure S19. Thermal-pulse amplitude (*ΔT*) under different actuation conditions. (a) *ΔT*–time profiles at different stretching amplitudes. (b) *ΔT*–time profiles at different stretching speeds. (c) *ΔT*–time profiles at different initial pre-strain levels (textile looseness).

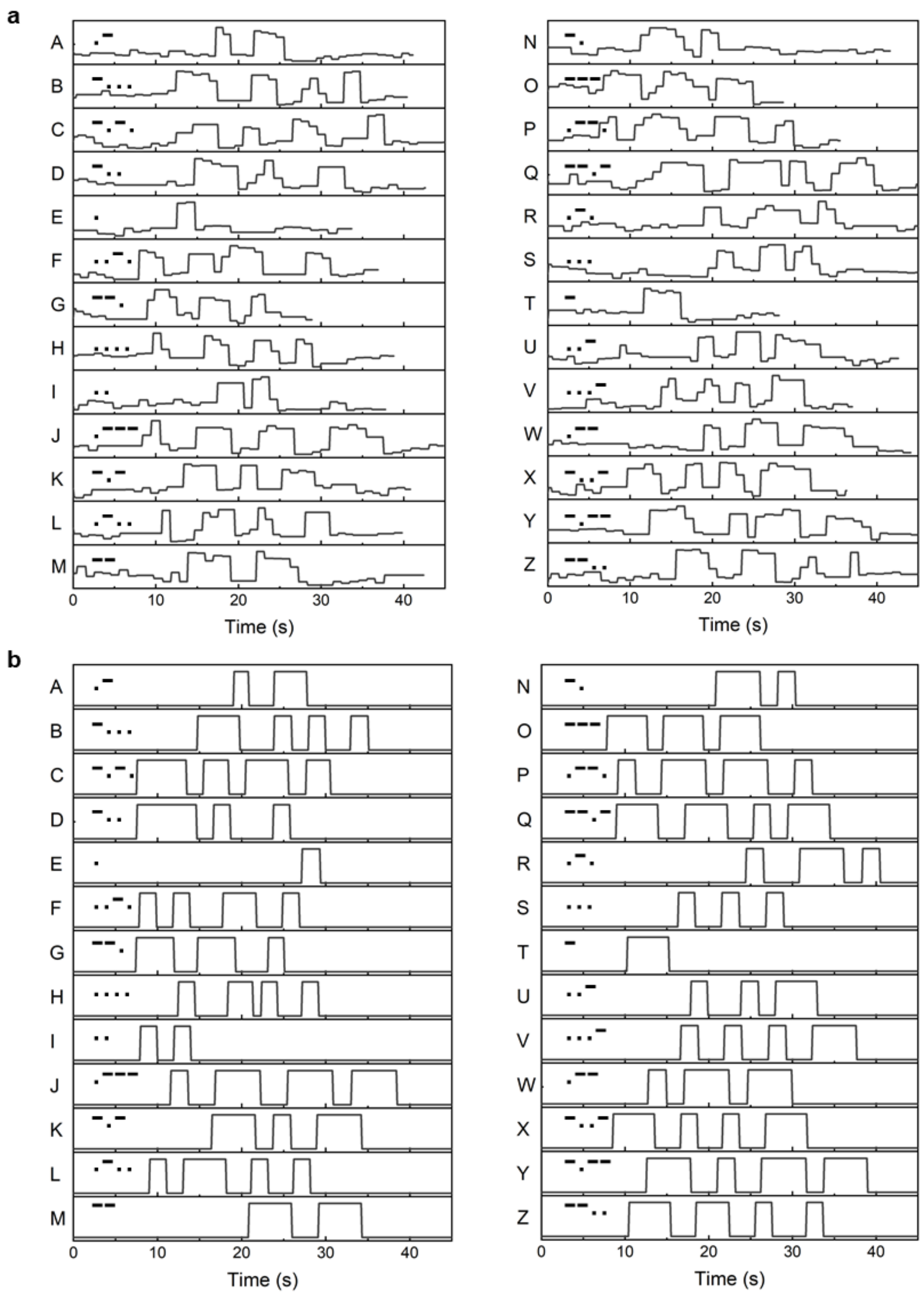

Figure S20. **Thermal signal outputs of the textile emitter representing Morse code letters.** (a, b) Corresponding signals of the 26 English letters before processing (a) and after processing (b).

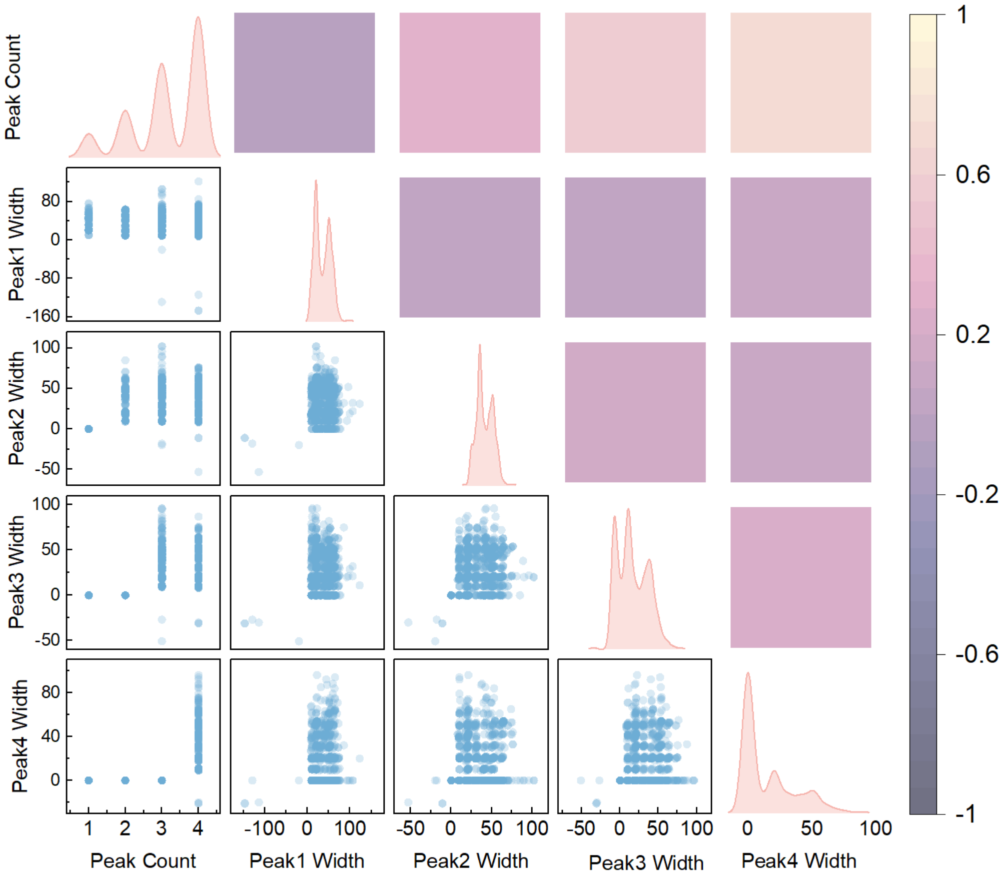

Figure S21. Feature scatter matrix and correlation heatmap of the standardized dataset. The scatter matrix illustrates the distributions and pairwise relationships between individual features, while the heatmap shows the corresponding linear correlation coefficients. The absence of strong linear correlations among features indicates limited feature redundancy within the dataset.

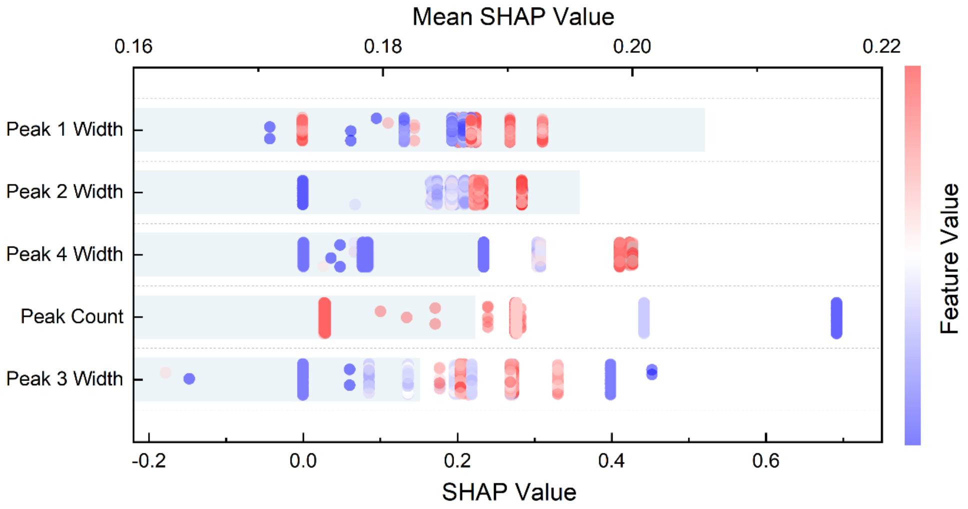

Figure S22. SHapley Additive exPlanations (SHAP) plot illustrating the ranked importance and individual contributions of each feature to model predictions. The feature importance ranking, from highest to lowest, is as follows: Peak 1 Width, Peak 2 Width, Peak 4 Width, Peak Count, and Peak 3 Width. SHAP values quantify each feature's influence on prediction outcomes, with larger absolute values indicating greater impact. The color of each point reflects the corresponding feature value in the sample, where red represents higher values and blue represents lower values.

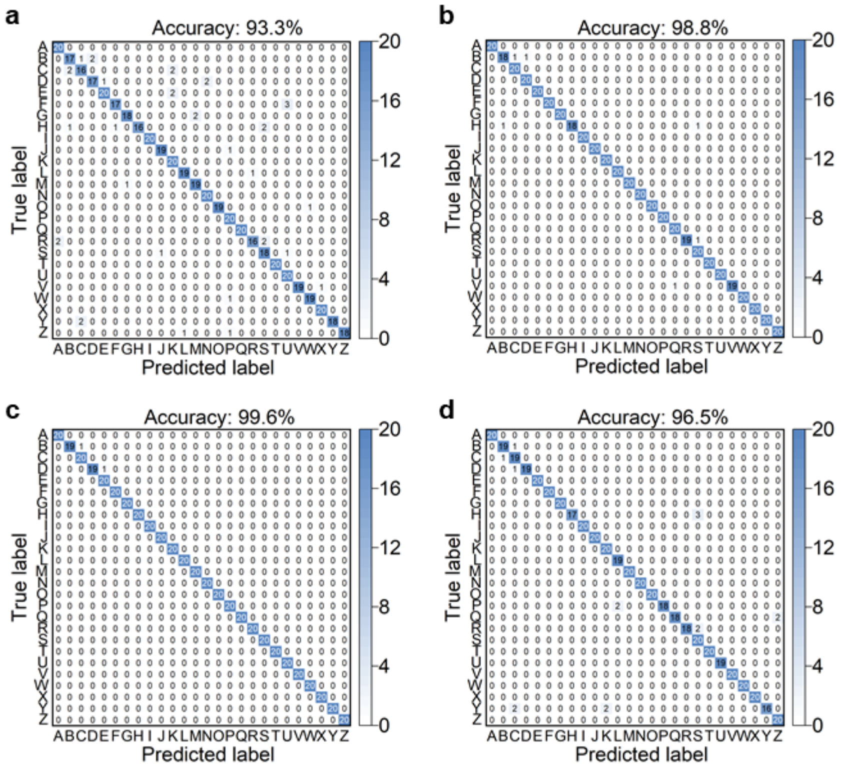

Figure S23. Confusion matrices of different machine learning algorithms for classifying 26 English letters. (a-d) Confusion matrices of KNN (a), SVM (b), RF (c), and XGBoost (d).

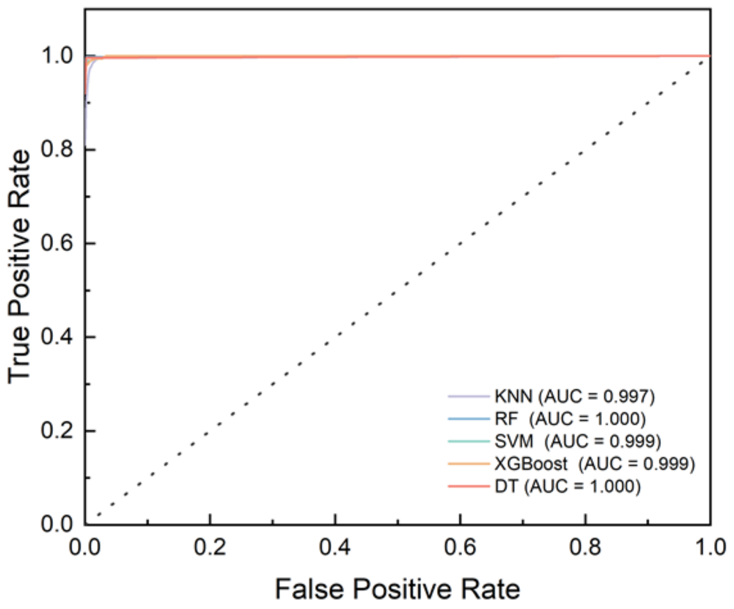

Figure S24. Area under the curve (AUC) for the classification of 26 English letters using five machine learning algorithms.

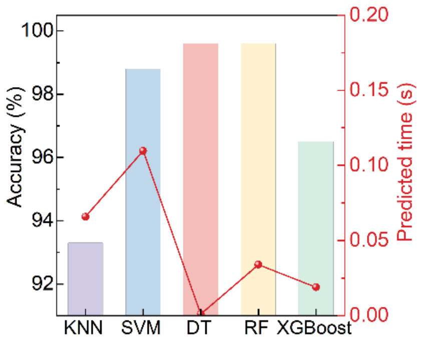

Figure S25. **Classification accuracy and computational time of five machine learning algorithms.**

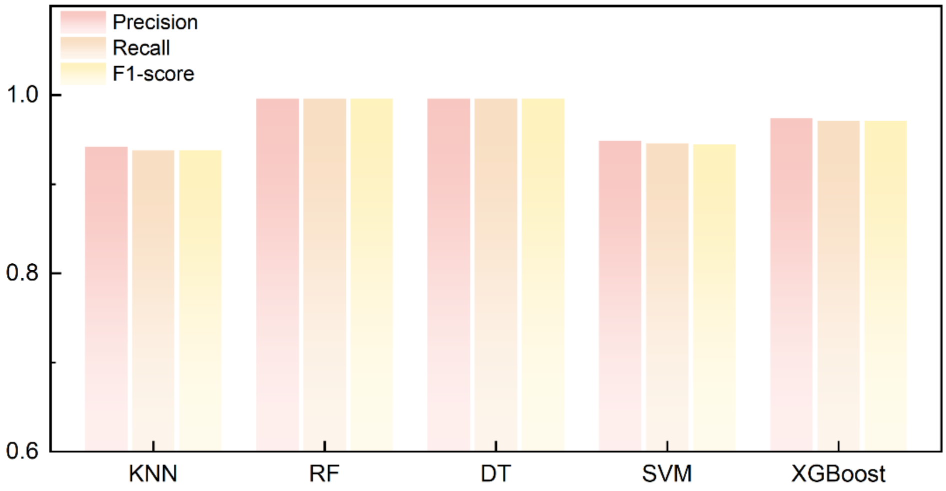

Figure S26. Precision, recall, and F1-score of five machine learning algorithm models for classifying 26 English letters.

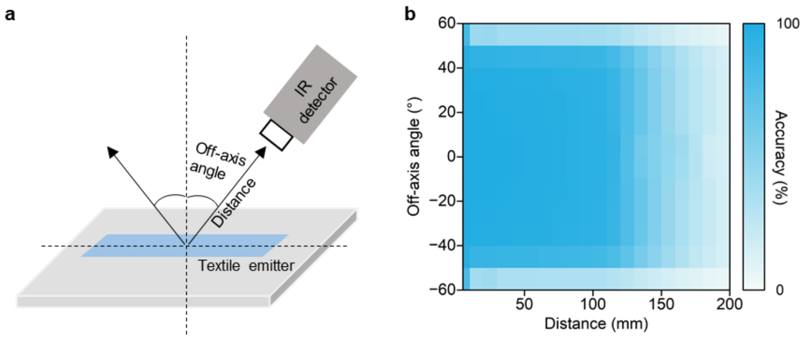

Figure S27. Recognition performance under different detector–emitter distances and field of view (FOV) conditions. (a) Schematic illustration of the experimental setup. (b) Recognition accuracy of short (‘dot’) and long (‘dash’) pulse signals as a function of detector–emitter distance and off-axis angle within the detector’s FOV.

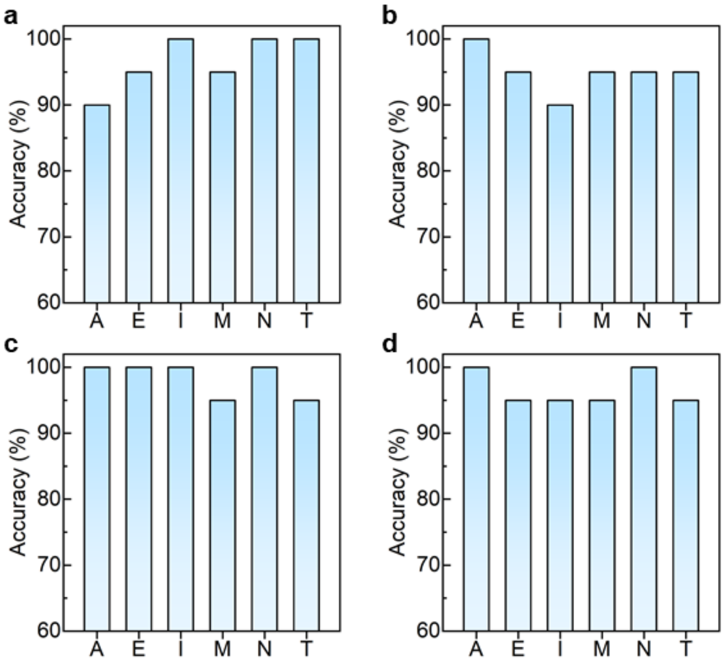

Figure S28. **Recognition performance of representative encoded commands under different ambient conditions.** (a) Strong solar irradiation (~1000 W m^{-2}). (b) High background radiation (ambient temperature >35 °C). (c) External heat-source interference. (d) Outdoor environments.

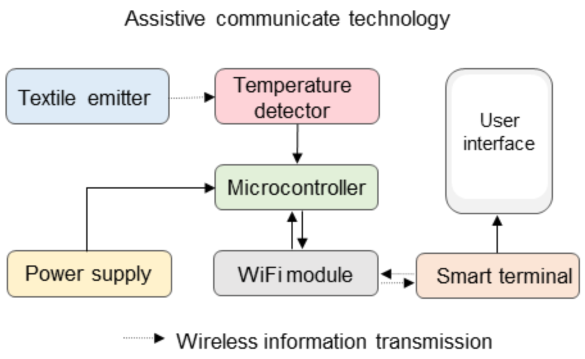

Figure S29. Block diagram of the textile emitter for information communication.

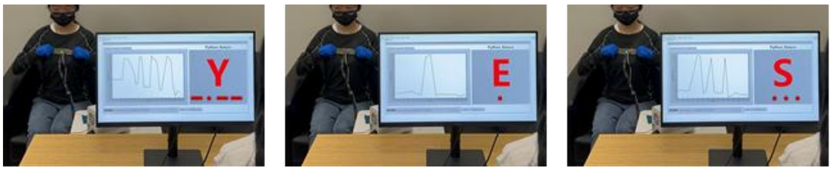

Figure S30. Demonstration of wireless information communication scenario based on the textile emitter, showing the transmission of the word 'YES'.

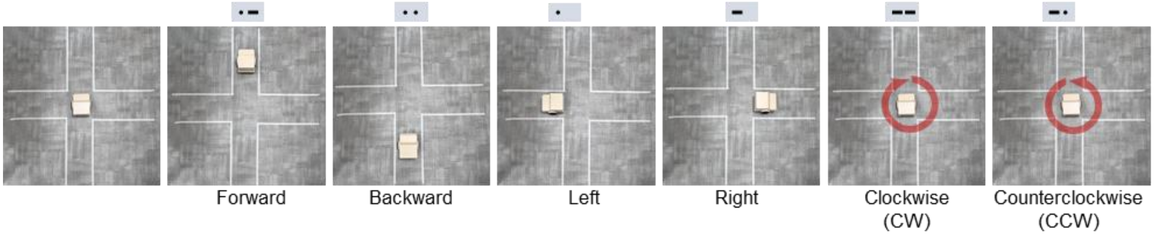

Figure S31. Signal characteristics and corresponding command definitions for robotic vehicle control.

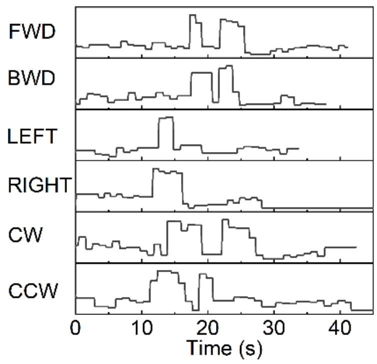

Figure S32. Raw thermal signal outputs of the textile emitter associated with six control commands: forward (FWD), backward (BWD), left, right, clockwise (CW), and counter-clockwise (CCW).

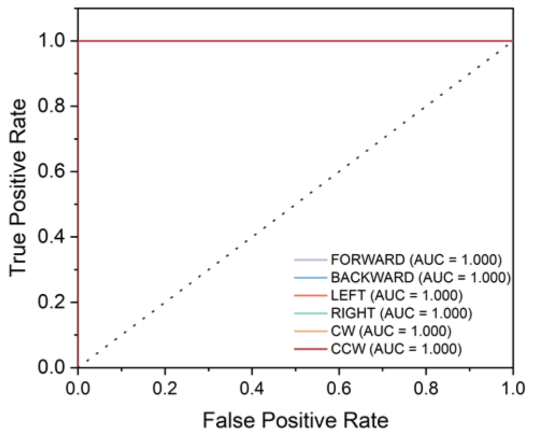

Figure S33. AUC values for the classification of six control commands using the DT algorithm.

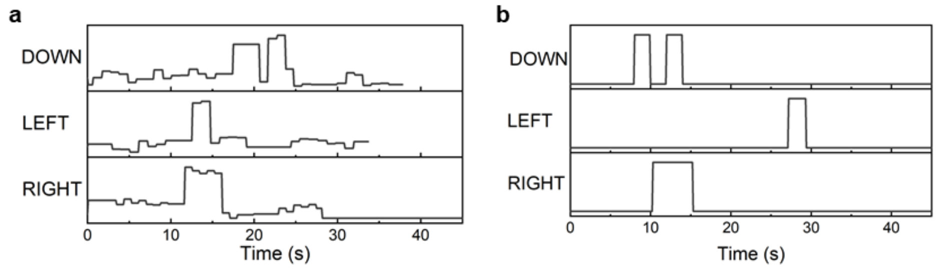

Figure S34. Thermal signal outputs associated with three control commands for digital game interaction: left, right, and down.

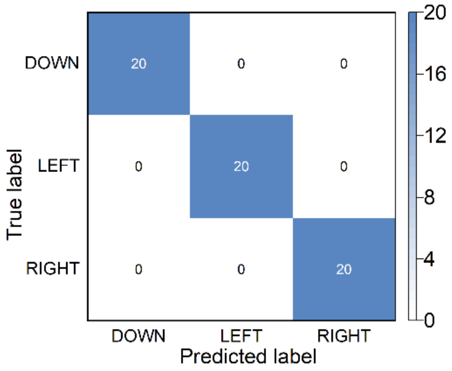

Figure S35. Confusion matrix of the DT algorithm for classifying three control commands.

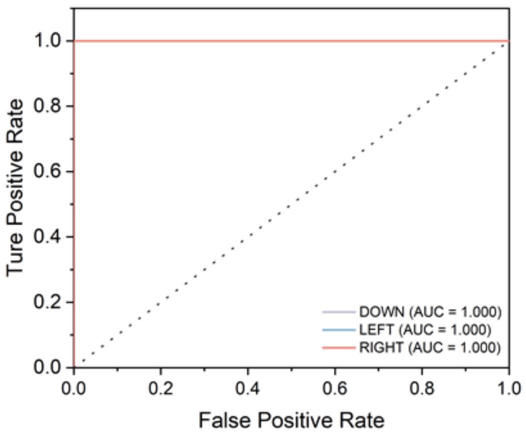

Figure S36. AUC values for classifying three control commands using the DT algorithm.

Table S1. Summary of representative interactive approaches and their key characteristics.

Approach	Signal modality	On-body electronics	Power / excitation requirement	Visual identity exposure	Wearability	References
Resistive textiles	Electrical	Yes	Continuous electrical power	No	Moderate	[1–4]
Capacitive textiles	Electrical	Yes	Continuous electrical power	No	Moderate	[5–8]
Triboelectric textiles	Electrical	Yes	Self-powered	No	Moderate	[9–12]
Piezoelectric textiles	Electrical	Yes	Self-powered	No	Moderate	[13–15]
Visible-light luminescent textiles	Optical	No	External excitation required (optical, electrical, or mechanical)	Low (externally observable)	Moderate	[16–19]
Camera-based systems	Optical (visible imaging)	No	Continuous electrical power	Yes	Not applicable	[20–21]
**This work**	**Thermal infrared emission**	**No**	**Passive (mechanically modulated)**	No	**High (textile-integrated, no on-body electronics)**	–

Note: In this work, thermal infrared radiation is intrinsically emitted by the textile, while mechanical deformation serves solely as a modulation mechanism for information encoding, rather than acting as an excitation or energy source.

Videos

Video S1. Demonstration of wireless information communication using the textile emitter, showing the transmission of the word ‘HI’.

Video S2. Demonstration of wireless information communication using the textile emitter, showing the transmission of the word ‘SOS’.

Video S3. Demonstration of wireless information communication using the textile emitter, showing the transmission of the word ‘YES’.

Video S4. Demonstration of wireless robotic vehicle control enabled by the textile emitter.

Video S5. Demonstration of wireless Tetris game control enabled by the textile emitter.

REFERENCES

1. Lu H, Zhang Y, Zhu M *et al.* Intelligent perceptual textiles based on ionic-conductive and strong silk fibers. *Nat Commun* 2024; **15**: 3289.
2. Jiang HJ, Underwood TC, Bell JG *et al.* A deep learning–enabled smart garment for accurate and versatile monitoring of sleep conditions in daily life. *Proc Natl Acad Sci* 2025; **120**: 2017.
3. Zhi Y, Zhang H, Zhang L *et al.* Pressure sensors based on densely structured graphene fibers for motion monitoring. *Adv Fiber Mater* 2025; **7**: 541–53.
4. Zhu W, Mo X, Wang Z *et al.* Machine learning-enhanced low-hysteresis conductive auxetic strain sensors with curved re-entrant honeycomb structures based on MXene/graphene for human rehabilitation training. *Chem Eng J* 2025; **505**: 159539.
5. Cheng A, Li X, Li D *et al.* An intelligent hybrid-fabric wristband system enabled by thermal encapsulation for ergonomic human-machine interaction. *Nat Commun* 2025; **16**: 591.
6. Lee J, Ihle SJ, Pellegrino GS *et al.* Stretchable and suturable fibre sensors for wireless monitoring of connective tissue strain. *Nat Electron* 2021; **4**: 291–301.
7. Fu C, Tang W, Miao Y *et al.* Large-scalable fabrication of liquid metal-based double helix core-spun yarns for capacitive sensing, energy harvesting, and thermal management. *Nano Energy* 2023; **106**, 108078.
8. Wu W, Jiang T, Wang M *et al.* Bioinspired monopolar controlled ionic hydrogels for flexible non-contact human–machine interfaces. *Adv Funct Mater* 2024; **34**: 2408338.
9. Ai Y, Wang Z, Liu Y *et al.* Robust fiber strain sensor by designing coaxial coiling structure with mutual inductance effect. *Adv Fiber Mater* 2024; **6**: 1629–39.
10. Lu X, Tan H, Zhang H *et al.* Triboelectric sensor gloves for real-time behavior identification and takeover time adjustment in conditionally automated vehicles. *Nat Commun* 2025, **16**: 1080.
11. Lin S, Yang W, Zhu X *et al.* Triboelectric micro-flexure-sensitive fiber electronics. *Nat Commun* 2024; **15**: 2374.

12. Shen Y, Chen C, Chen L *et al.* Nano energy mass-production of biomimetic fur knitted triboelectric fabric for smart home and healthcare. *Nano Energy* 2024; **125**: 109510.
13. Wang K, Sun X, Cheng S *et al.* Multispecies-coadsorption-induced rapid preparation of graphene glass fiber fabric and applications in flexible pressure sensor. *Nat Commun* 2024; **15**: 5040.
14. Yin H, Li Y, Tian Z *et al.* Ultra-high sensitivity anisotropic piezoelectric sensors for structural health monitoring and robotic perception. *Nano-Micro Lett* 2025; **17**: 42.
15. Luo Y, Li Y, Sharma P *et al.* Learning human–environment interactions using conformal tactile textiles. *Nat Electron* 2021; **4**: 193-201.
16. Li P, Wang Y, He X *et al.* Wearable and interactive multicolored photochromic fiber display. *Light Sci Appl* 2024; **13**: 48.
17. Yang W, Gong W, Gu W *et al.* Self-powered interactive fiber electronics with visual–digital synergies. *Adv Mater* 2021; **33**: 2104681.
18. He J, Wei R, Ma X *et al.* Contactless user-interactive sensing display for human–human and human–machine interactions. *Adv Mater* 2024; **36**: 2401931.
19. Chen YT, Wang HL, Sun S *et al.* Computer-vision based gesture-metasurface interaction system for beam manipulation and wireless communication. *Adv Sci* 2024; **11**: 2305152.
20. Ma Q, Gu Z, Gao X *et al.* Intelligent hand-gesture recognition based on programmable topological metasurfaces. *Adv Funct Mater* 2025; **35**: 2411667.
21. Chaaraoui AA, Padilla-López JR, Ferrández-Pastor FJ *et al.* A vision-based system for intelligent monitoring: Human behaviour analysis and privacy by context. *Sensors* 2014; **14**: 8895–925.
